# Line-field confocal optical coherence tomography coupled with artificial intelligence algorithms to identify quantitative biomarkers of facial skin ageing

**DOI:** 10.1038/s41598-023-40340-0

**Published:** 2023-08-24

**Authors:** Franck Bonnier, Mélanie Pedrazzani, Sébastien Fischman, Théo Viel, Agnes Lavoix, Didier Pegoud, Meryem Nili, Yolande Jimenez, Samuel Ralambondrainy, Jean-Hubert Cauchard, Rodolphe Korichi

**Affiliations:** 1 LVMH Recherche, 185 Avenue de Verdun, 45804 Saint Jean de Braye, France; 2 DAMAE Medical, 14 Rue Sthrau, 75013 Paris, France; 3 DERMATECH, 8 Rue Jacqueline Auriol, 69008 Lyon, France

**Keywords:** Three-dimensional imaging, Ageing, Optical imaging, 3-D reconstruction, Confocal microscopy

## Abstract

Quantitative biomarkers of facial skin ageing were studied from one hundred healthy Caucasian female volunteers, aged 20–70 years, using in vivo 3D Line-field Confocal Optical Coherence Tomography (LC-OCT) imaging coupled with Artificial Intelligence (AI)-based quantification algorithms. Layer metrics, i.e. stratum corneum thickness (SC), viable epidermal thickness and Dermal–Epidermal Junction (DEJ) undulation, as well as cellular metrics were measured for the temple, cheekbone and mandible. For all three investigated facial areas, minimal age-related variations were observed in the thickness of the SC and viable epidermis layers. A flatter and more homogeneous epidermis (decrease in the standard deviation of the number of layers means), a less dense cellular network with fewer cells per layer (decrease in cell surface density), and larger and more heterogeneous nuclei within each layer (increase in nuclei volume and their standard deviation) were found with significant variations with age. The higher atypia scores further reflected the heterogeneity of nuclei throughout the viable epidermis. The 3D visualisation of fine structures in the skin at the micrometric resolution and the 1200 µm × 500 µm field of view achieved with LC-OCT imaging enabled to compute relevant quantitative biomarkers for a better understanding of skin biology and the ageing process in vivo.

## Introduction

Visible signs of ageing such as skin sagging, wrinkles, sunspots, and uneven skin colour define an individual’s perceived age to others and also their own body image^1^. Although, atlases can be used for grading the severity of macroscopic clinical signs^2^, skin ageing results from the accumulation of damages at the cellular and molecular levels over time, exacerbated by intrinsic (genetics, cellular metabolism, hormonal and metabolic processes) and extrinsic (chronic light exposure, pollution, chemicals, …) factors that make the correlation between the physiological mechanisms and the visible effects difficult.

While torsion or suction devices are used to investigate in vivo age-related changes in mechanical properties^3^, non-invasively monitoring and quantifying changes occurring below the skin surface in underlying microstructures of the epidermis and dermis is challenging. High-Frequency Ultrasound (HFUS) imaging at 22–75 MHz is limited to the investigation of the dermis and properties of the Subepidermal Low-Echogenic Band (SLEB)^4^. Optical Coherence Tomography (OCT), a widespread technique for ophthalmology^5^, cardiology^6^, gastroenterology^7^ or dermatology (diagnosis of skin lesions)^8^, does not enable to characterise the fine skin structures of the viable epidermis (VE) and stratum corneum (SC) due to limited axial resolution (~ 10 μm)^9^.

Optical microscopy techniques, such as Confocal Laser Scanning Microscopy (CLSM)^10,11^, or Multiphoton Laser Scanning Microscopy (MPLSM)^12^ can achieve higher lateral and axial resolutions when observing micrometric-level features of superficial skin layers. Nevertheless, the laser sources and focusing optics commonly employed in these techniques impose limitations on penetration depth, typically around 250 μm^13^. Recent instrumental and data analysis improvements have contributed to quantifying, i.e. correlating, structural modifications with ageing through the automation of in vivo imaging protocols^14,15^, but access to live 3D visualisation of the skin remains to be addressed. Line-field Confocal Optical Coherence Tomography (LC-OCT) is an emerging imaging technique^16^ that extends the principles of time-domain OCT (TD-OCT)^17^. With LC-OCT, it is possible to achieve a field-of-view of 1200 μm × 500 μm × 500 μm during in vivo 3D imaging, with a lateral resolution of approximately 1 µm, and an acquisition time within seconds^16^. Initially developed for acquiring ultra-high resolution vertical section images (B-scans)^18^, acquisitions of both vertical (B-scan) and *en face* (horizontal) section images (C-scans) has provided isotropic high resolution 3D LC-OCT images^19^ adapted to study skin histological and cellular structures at the micrometric level^20,21^. Coupled with Artificial Intelligence (AI)-based segmentation algorithms, the technique is a promising method to investigate skin disorder such as pustular skin^22^ or Actinic Keratosis^23^ but also to derive 3D quantitative parameters from healthy skin to study the intracutaneous effects of ageing^24,25^.

The present work reports the first study conducted on 100 healthy Caucasian female volunteers (20–70 years old) to identify biomarkers of facial skin ageing using LC-OCT 3D imaging. Current confocal microscopic imaging techniques do not enable full-face analysis hence most studies have targeted specific anatomical sites to study aging-related features in each region^26^. In this study, three sites have been selected: one in the upper part of the face (temple), one in the central (malar) region (cheekbone), and one in the lower part (inferior jawline). These sites aim to capture changes from three distinct and representative compartments across the entire face. Images acquired from three facial regions (temple, cheekbone, and mandible) were subjected to AI-assisted analysis, enabling the computation of metrics (parameters) for the thickness of skin layers, including the stratum corneum (SC) and the viable epidermis (VE). Additionally, metrics were obtained for cell morphology, specifically nuclei size and shape, as well as cell network atypia, which represents a multiparametric score derived from comparing the shape and size of nuclei with neighbouring cells^25^. Furthermore, the quantitative cellular metrics were analysed with respect to their depth within the viable epidermis to account, to some extent, for the biological variations occurring during the maturation process of keratinocytes, encompassing the *Stratum Basale*, *Stratum Spinosum*, and *Stratum Granulosum* layers.

## Results and discussion

### Histological metrics

The thickness of the SC*,* the thickness of the VE and the undulation of the DEJ were determined for the temple, cheekbone and mandible (Table 1). The corresponding boxplots are provided as supplementary materials (Figs. S1, S2 and S3).

**Table 1:** SC thickness, viable epidermis thickness and DEJ undulation determined for the temple, cheekbone and mandible.

Parameters		[20,30]	[31,40]	[41,50]	[51,60]	[61,70]	Test
Temple							
SC thickness (µm)	Mean (sd)	13.2 (0.8)	13.4 (0.8)	13.4 (1.2)	13.6 (0.9)	14.2 (1.0)	*p* = 0.0039 (Kruskal Wallis)
	Mult. comp	a	ab	ab	ab	b	
Viable epidermis thickness (µm)	Mean (sd)	54.6 (6.8)	53.3 (4.6)	51.4 (3.9)	53.0 (4.9)	52.1 (4.8)	*p* = 0.42 (Kruskal Wallis)
	Mult. comp	–	–	–	–	–	
Undulation DEJ	Mean (sd)	2.64 (2.04)	2.31 (1.76)	2.12 (1.88)	2.42 (1.96)	2.07 (1.26)	*p* = 0.92 (Kruskal Wallis)
	Mult. comp	–	–	–	–	–	
Cheekbone							
SC thickness (µm)	Mean (sd)	12.1 (0.5)	12.4 (0.5)	12.5 (0.9)	12.3 (0.6)	12.5 (0.8)	*p* = 0.31 (Kruskal Wallis)
	Multi. Comp	–	–	–	–	–	
Viable epidermis thickness (µm)	Mean (sd)	48.4 (6.5)	49.0 (6.6)	44.7 (7.8)	46.8 (6.1)	45.7 (7.4)	*p* = 0.26 (Kruskal Wallis)
	Mult. comp	–	–	–	–	–	
Undulation DEJ	Mean (sd)	0.26 (0.39)	0.46 (0.56)	0.37(0.42)	0.475 (0.457)	0.48 (0.48)	*p* = 0.13 (Kruskal Wallis)
	Mult. Comp	–	–	–	–	–	
Mandible							
SC thickness(µm)	Mean (sd)	13.1 (0.4)	13.3 (1.0)	13.4 (0.8)	13.6 (0.8)	13.9 (1.3)	*p* = 0.04 (Kruskal Wallis)
	Mult. comp	a	ab	ab	ab	b	
Viable epidermis thickness (µm)	Mean (sd)	55.1 (7.2)	57.5 (8.2)	51.0 (4.6)	50.5 (6.4)	48.1 (5.3)	*p* = 0.0003 (Kruskal Wallis)
	Mult. comp	bc	c	ab	ab	a	
Undulation DEJ	Mean (sd)	1.44 (1.66)	1.19 (1.13)	1.42 (1.52)	1.44 (1.04)	1.08 (0.84)	*p* = 0.92 (Kruskal Wallis)
	Mult. Comp	–	–	–	–	–	

The mean SC thickness for the temple displayed a 7.6% increase according to age groups from 13.2 ± 0.8 µm (age group [20, 30]) to 14.2 ± 1.0 µm (age group [61,70]) (KW *p* = 0.0039) (Table 1). The pairwise comparison indicated that age groups [61,70] and [20, 30] as statistically different. For the mandible a comparable pattern was observed with means equal to 13.1 ± 0.4 µm (age group [21, 30]) and 13.9 ± 1.3 µm (age group [61,70]) (KW *p* = 0.04), corresponding to a 6.1% increase that correlates with age groups according to the pairwise comparison. For the cheekbone, age-related variations were not found to be significant (KW *p* = 0.31) (Table 1). Although, for the mandible, the VE exhibited a thinning with age of 12.7% from 55.1 ± 7.2 µm (age group [20, 30]) to 48.1 ± 5.3 µm (age group [61,70], KW *p* = 0.0003), the temple and the cheekbone did not show any significant change (Table 1). The undulation of the DEJ was also determined for each age group but no evolution with age could be highlighted (KW > 0.1). The three facial areas studied displayed fairly consistent values, i.e. ~ 2.50% for the temple, ~ 0.50% for the cheekbone and ~ 1.20% for the mandible. Mean values under 3% presently found suggested the DEJ is relatively flat for the youngest volunteers hence the limited variations observed.

Monnier et al. (2020) investigated the potential of vertical 2D LC-OCT imaging for in vivo characterization of healthy human skin, comparing different body sites to determine the thickness of the SC and the total epidermis^27^. Data from different anatomical sites of the face in 10 healthy volunteers (mean age = 27.1 ± 5.2 years) were analysed. When comparing the age group [20, 30], the thickness of the SC in the hollow of the cheek equal to 9 ± 1.1 µm, was found lower than the thickness presently found for the cheekbone, which was 12.1 ± 0.5 µm. However, the thickness of the viable epidermis for the hollow of the cheek (~ 49 µm) was comparable to the mean value of 48.4 ± 6.54 µm found for the cheekbone. In 2021, Chauvel-Picard et al. proposed a pilot study on the characterization of healthy epidermis, coupling 3D LC-OCT imaging with AI-assisted computing of quantitative metrics. Similar variations in seven body sites were also observed, with respective thicknesses of 9.7 ± 1.6 µm for the SC and 49.7 ± 4.2 µm for the viable epidermis (total epidermis thickness minus SC thickness) for the cheek^24^. A difference of ~ 2 µm for the SC compared to the present results is observed; however, the correlation of results is limited due to the unbalanced number of volunteers (pilot study n = 5, present study n = 100). Importantly, the work by Chauvel-Picard et al. highlighted the variability that can be witnessed among different anatomical sites of the face. Notably, the comparison of the forehead (SC = 11.6 ± 2.5 µm; epidermis = 69.4 ± 11.87 µm), the nose (SC = 13.0 ± 3.4 µm; epidermis = 76.4 ± 9.6 µm), and the hollow of the cheek (SC = 9.7 ± 1.6 µm and epidermis = 49.7 ± 4.2 µm) showed significant variations in the thickness of superficial layers. It is likely that the hollow of the cheek (soft part) and the cheekbone (below the eye socket), which are a few centimetres apart, display noticeable differences. Interestingly, a comparative study of the SC thickness measured in vivo with confocal Raman spectroscopy (CRS) and CRM has reported a mean of 12.8 ± 1.5 µm for the cheek (n = 17 volunteers)^28^. This observation was confirmed in two other studies, the first comparing CRS with OCT for the measurement of the SC thickness (mean = 12.8 ± 0.9 µm)^29^, and the second investigating the variations in normal skin using CRM (mean = 12.05 ± 1.7 µm)^30^. These observations confirm that the automated segmentation performed presently delivered reliable estimations for the SC thickness that are consistent with the literature. Regarding the thickness of the epidermis, a systematic review coupled with a meta-analysis allowed for screening results collected using OCT, ultrasounds, LSM, and histology to compute mean skin thickness for various body sites^26^. For instance, from the literature data, a mean epidermal thickness equal to 56.5 µm for the cheek of female Caucasians was obtained, which is consistent with the means calculated from the LC-OCT results.

Regarding the effects of aging, initial work by Shuster et al. in 1975 on human biopsies reported that for women, the skin total thickness was constant until around the age of 50–60 years after which it decreased slightly^31^. Since then, in vivo technologies for dermatological research have evolved to enable quantification of ageing effects. It is generally accepted that the SC maintains its thickness during ageing^32^, the epidermis is expected to thin significantly with age and the rete ridges to flatten^32^. Despite histological evidence^33^, changes in dermis, epidermis, and whole skin thickness measured in vivo reported in the literature are often contradictory^34–36^. HFUS has gained significant recognition as a reliable technique for investigating variations in the SLEB^37^, It has also been extensively used in studies that primarily focus on non-invasive measurements of skin thickness in vivo. Recent in vivo studies reporting a decrease in total skin thickness on the dorsal forearm and ventral thigh^34^; the effects of the photo-ageing at the dermis level for different areas of the forearm^35^; and the absence of difference in cheek epidermal thickness (among others body sites) between two age groups < 35 years old and > 35 years old^38^, all suggested that the main variations in skin thickness with age are due to changes in the deepest layers of the skin. A thicker total skin in young individuals and a decrease for very mature skin age^4^ are probably the main features established from HFUS investigations to date. The higher resolving power of optical microscopy techniques has enabled a more refined analysis, but the results remain sometimes difficult to corroborate. A multiparametric quantification of human skin ageing on the forearm and face using 3D multiphoton imaging concluded that a thinning of the epidermis explained most of the difference between age groups (i.e. 18–25 years, n = 15 versus 70–75 years, n = 15) but no significant variations were found in the SC in European female volunteers^39^. However, no age-related differences were observed by RCM in the malar region for SC and VE (age groups 18–35 years, n = 6 and 40–60 years, n = 6)^40^. Similarly, a multiphoton laser tomography (MPT) study on the forearm and hand has showed no difference in VE and SC thickness between 3 age groups (mean age groups = 23.3, 47.3 and 72.1 years, n = 10 each)^12^. The same conclusions were drawn for the assessment of chronological and photo ageing of the forearm skin using RCM (age group 20–30 years: 9 males, 28 females; age group 50–60 years: 24 males, 14 females)^41^. There is undoubtedly a lack of standardisation in experimental designs studying different body sites and panels (ethnicities, phototypes, age groups). The panels studied are also generally too small to reach a level of confidence in the quantification of age-related modifications. However, the acquisition speed of LC-OCT 3D imaging (< 1 min for a full 3D stack), the field of view (1200 µm × 500 µm) and the micrometric resolution open up perspectives to generate valuable in vivo data from large cohorts of volunteers. Presently, the investigation conducted on 100 volunteers aimed at providing further insights into age-related variations.

Firstly, the results collected using LC-OCT imaging enabled to emphasise a thickening of the SC (~ + 1 µm) between age groups [20–30] and [61–70]. A more comprehensive analysis of the results reported by Koehler et al.^12^, utilizing confocal laser tomography on the dorsal forearm, revealed the following SC thickness values: 18 µm (Group #1, mean age = 23.3 years), 16 µm (Group #2, mean age = 47.3 years), and 25 µm (Group #3, mean age = 72.1 years). Despite the differences were not found to be statistically significant, an increase in mean values of 7 µm was observed. The data reported by Pena et al.^39^, utilizing MPM, exemplifies the challenge in reaching a consensus about SC thickness across different age groups. Comparing two separate studies conducted on the ventral forearm, the first study from 2010 showed an increase in mean thickness from 16.64 ± 2.81 µm (age group [30–40]) to 18.44 ± 5.03 µm (age group [55–65]), while the second study conducted in 2009 on ventral and dorsal forearms showed a decrease from 15.80 ± 2.83 µm to 13.33 ± 3.50 µm on the ventral forearm and an increase from 13.93 ± 3.86 to 15.65 ± 4.93 µm on the dorsal forearm for age groups [18–25] and [70–75], respectively. Although these findings were not statistically significant, the presented box plots clearly displayed trends in data distributions that appeared to correlate, at some extend, with aging. Interestingly, a third study included acquisitions performed on the temple, resulting in SC thickness measurements of 11.06 ± 2.72 µm (age group [18–25]) and 11.55 ± 2.56 µm (age group [70–75]), with only a 0.5 µm difference. In comparison with present LC-OCT results, it was found that for the temple, the means increased from 13.2 ± 0.8 µm for age group [20–30] to 14.2 ± 1 µm for age group [60–70]. The same pattern and a variation in the same order of magnitude were observed, but the main difference lies in the standard deviations, which were lower for LC-OCT (SD ~ 1), yielding statistically significant different results. Measuring the stratum corneum (SC) thickness in vivo inevitably raises questions about the performance of the technique. There are limited studies reporting data collected from the face using technologies with adequate resolution and robustness (repeatability, reproducibility) to accurately assess non-invasively the effects of aging on the SC thickness in vivo. Powerful methods, such as LC-OCT 3D imaging, play a crucial role in quantifying micrometric variations and highlighting subtle changes that were previously not discernible or disregarded due to technical limitations.

Secondly, the current LC-OCT investigation has brought to light that the viable epidermis VE does not consistently undergo thinning with age. While the mandible displayed a decrease of approximately 7 µm, the cheekbone and temple showed no noticeable variations, indicating differences among the studied areas. Moreover, a pilot study employing LC-OCT^24^ reported no significant difference in SC and VE thickness between age groups (24.2 ± 2.4 years (n = 5) and 57.0 ± 1.0 (n = 3)) for the cheek, nose, and forehead. However, the current work represents the first extensive study (n = 100) examining this aspect with this method. The topic of the variation in viable epidermis thickness remains somewhat controversial within the scientific community. This can be attributed to numerous factors that affect the interpretation of results, including variations in sample sizes, demographics, and study designs. Additionally, other factors, such as genetics, environmental influences, or underlying skin conditions, contribute to conflicting findings, making it challenging to draw definitive conclusions about viable epidermal thinning and its association with aging. This is evident in the systematic review and meta-analysis conducted by Lintzeri et al.^26^, which examined epidermal thickness in healthy humans. Out of 142 studies spanning from June 1946 to June 2020 and containing sufficient information, only 9 studies were related to facial skin aging. Among these, one study used histology on biopsies from Korean volunteers^42^, five studies used OCT, involving Caucasians^43–45^, Africans^45^, and Asians^46^, and three studies employed RCM, including populations of Caucasians^47^, Koreans^48^, and Brazilians^49^. In addition to the variation in panels size and ethnic groups studied, it is essential to emphasize that the studies by Josse et al.^43^, Querleux et al.^45^ and Pouradier et al.^44^ did not provide direct data for age group comparisons (i.e. only one age group studied). Instead, the results of epidermis thickness were used to estimate mean values for young and old groups. Conducting in-depth analysis of the different studies, considering the techniques used, body sites studied, ethnic groups, or phototypes, it becomes challenging to draw a conclusive statement regarding the systematic thinning of the viable epidermis with aging for the face. The age range selected for the investigation is an important criterion to consider for the interpretation of the results. The study by Kim et al. conducted on the cheek found no differences in epidermis thickness with aging between young and old age groups, defined as [20–28 years old] and [50–58 years old], respectively^48^. Comparing these findings, the conclusion from Longo et al. study on the malar eminence^47^, suggesting a difference in epidermal thickness caused by aging, might initially seem contradictory. However, when comparing results based on age groups, no statistical differences were observed between the four age groups: [< 35 years old], [36–45 years old], [46–55 years old], and [56–65 years old]. A noticeable decrease in thickness was observed only for the age group [65–82 years old]. This observation was further emphasised in another study by Longo et al., which reported a significant difference for the same age group [65–82 years old]^10^. The results obtained through LC-OCT imaging in the present study corroborate the previously reported consistent thickness of the viable epidermis up to 65 years old, as observed with RCM. Unfortunately, due to differing inclusion criteria, the older age group [61–70 years old] did not allow to observe further evolution, i.e., potential thinning, for volunteers over 70 years old. Nevertheless, the quantification of skin microstructures using LC-OCT 3D imaging has demonstrated its potential to enhance the current knowledge about age-related structural variations in facial skin. It is a relevant tool to consider for supporting the establishment of a consensus within the community regarding the definition of normal aging features.

### Cellular metrics

The number of cell layers computed for the three regions are presented in Table 2. For the mandible, the mean decreased from 6.28 ± 0.65 layers (age group [20, 30]) to 5.64 ± 0.57 layers (age group [60,70]) (*p* = 0.002). The standard deviation of the number of cell layers also had a significant decrease from 1.19 ± 0.12 to 1.05 ± 0.11 layers (*p* < 0.001). The pairwise test discriminated between the youngest and oldest age groups, suggesting that for the age group [20, 30] there is a higher heterogeneity in the number of cell layers. While the observed decrease of ~ 7 µm in the epidermis thickness for the mandible correlates with the lower number of cell layers, the decreased standard deviation indicated a flatter and more homogeneous aspect. For the temple and cheekbone, the number of cell layers did not show significant differences, although for the temple standard deviation of the number of cell layers confirmed a higher heterogeneity for the youngest group (*p* = 0.038).

**Table 2:** Number of cell layers and standard deviation for the number of cell layers determined for the temple, the cheekbone and the mandible.

Parameters		[20,30]	[31,40]	[41,50]	[51,60]	[61,70]	Test
Temple							
Layer number	Mean (sd)	6.16 (0.58)	6.02 (0.48)	5.86 (0.56)	5.88 (0.57)	5.87 (0.50)	*p* = 0.412 (Kruskal Wallis)
	Mult. comp	–	–	–	–	–	
Standard deviation layer number	Mean (sd)	1.28 (0.18)	1.24 (0.11)	1.17 (0.09)	1.21 (0.11)	1.14 (0.12)	*p* = 0.038 (Kruskal Wallis)
	Mult. comp	b	ab	ab	ab	a	
Cheekbone							
Layer number	Mean (sd)	5.37 (0.63)	5.59 (0.71)	5.25 (0.80)	5.34 (0.51)	5.37 (0.75)	*p* = 0.623 (ANOVA)
	Mult. comp	–	–	–	–	–	
Standard deviation layer number	Mean (sd)	1.10 (0.14)	1.10 (0.16)	1.01 (0.11)	1.06 (0.12)	1.03 (0.13)	*p* = 0.118 (ANOVA)
	Mult. comp	–	–	–	–	–	
Mandible							
Layer number	Mean (sd)	6.28 (0.65)	6.39 (0.61)	6.04 (0.52)	5.76 (0.65)	5.64 (0.57)	*p* = 0.002 (Kruskal Wallis)
	Mult. comp	bc	c	abc	ab	a	
Standard deviation layer number	Mean (sd)	1.19 (0.12)	1.22 (0.13)	1.11 (0.14)	1.10 (0.13)	1.05 (0.11)	*p* < 0.001 (Kruskal Wallis)
	Mult. comp	bc	c	abc	ab	a	

In Tables 3, 4 and 5 the cellular metrics are presented according to 5 maturation indexes resulting from the segmentation of the VE. M1 is the deepest segment of the VE corresponding to the *stratum basale* and M5 is the last segment assimilable to the *stratum granulosum*. *Cell surface density* (CSD) was found to significantly decrease with age for the temple (Table 3) at M5 (*p* = 2.34e^−7^), M4 (*p* = 3.99e^−5^) and M3 (*p* = 0.012); for the cheekbone (Table 4) at M5 (*p* = 0.0018) and M4 (*p* = 0.0185); and for the mandible (Table 5) at M5 (*p* = 1.23e^−5^), M4 (*p* = 1.70e^−8^) and M3 (*p* = 1.75e^−5^). For the 3 facial areas, CSD in the *stratum granulosum* and *stratum spinosum* (M5–M3) displayed age-related variations. The decrease in CSD highlighted a less dense cellular network with reduced number of cells per layer. *The mean nuclei volumes* for the temple (Table 3), the cheekbone (Table 4) and the mandible (Table 5) increased with age groups for all maturation indexes. However, significant variations were found for the mandible at M5 (*p* = 0.0019), M4 (*p* = 0.017) and M1 (*p* = 0.0164) and for the temple at M2 (*p* = 0.0437) and M1 (*p* = 0.0373), all of which also discriminated between age groups [20, 30] and [61,70]. The *standard deviation of nuclei volume* exhibited a significant increase for the temple (Table 3) at M5 (*p* = 0.0466) and M4 (*p* = 0.0124); the cheekbone (Table 4) at M5 (*p* = 0.0128), M4 (*p* = 0.0302), M3 (*p* = 0.0396) and M2 (*p* = 0.019); and for the mandible (Table 5) at M5 (*p* = 0.0002), M4 (*p* = 0.0001), M3 (*p* = 0.0087), M2 (*p* = 0.006) and M1 (*p* = 0.0295). Pairwise comparisons highlighted significant age-related differences for the cheekbone and mandible at a depth corresponding to the outer part of the VE, i.e. the *stratum granulosum* and *stratum spinosum* (maturation indexes M5–M3). The increase in mean nuclei volume and its standard deviation indicated the presence of larger and more heterogenous nuclei in these layers. Despite significant differences *in nuclei compactness* for the temple (Table 3), at M4 (*p* = 0.012); for the cheekbone (Table 4) at M4 (*p* = 0.0011), M3 (*p* = 6.250e^−7^), M2 (*p* = 1.195e^−5^) and M1 (*p* = 0.0032); and for the mandible (Table 5) at M4 (*p* = 0.0096), M3 (*p* = 0.0002) and M2 (*p* values = 0.037), pairwise comparisons only confirmed a decrease between the [20, 30] and [61,70] age groups for the cheekbone. The *standard deviation of compactness* also showed a significant increase in heterogeneity associated with ageing for the M3 (*p* = 1.680e^−5^), M2 (*p* = 2.355e^−6^) and M1 (*p* = 0.0122) maturation indexes for the cheekbone. *Cell network atypia* displayed a significant increase for the temple (Table 3) for M5 to M1 (*p* < 0.01); for the cheekbone (Table 4) for M5 to M2 (*p* < 0.05); and for the mandible (Table 5) for M5 to M2 (*p* < 0.05). Pairwise comparisons confirmed that age-related variations can be found in the cell network atypia metric at different depths of the VE for the temple and the mandible while for the cheekbone the discrimination was achieved for maturation indexes corresponding to the *stratum granusolum* (M5–M4). Cell network atypia is a multiparametric cellular metric taking into account the nuclei shape and size as well as the comparison to direct neighbouring nuclei to compute the score^25^. Therefore, the score is a relevant quantitative marker of nuclei heterogeneity that revealed substantial variations with age for the 3 facial areas studied.

**Table 3:** Summary of cellular metrics as function of maturation index (Temple).

Parameters		M5	M4	M3	M2	M1
CSD (cells/mm^2^)	[20,30]	2934 ± 195 (c)	5368 ± 365 (c)	5919 ± 481 (b)	4851 ± 551	2996 ± 507
	[31,40]	2823 ± 167 (bc)	5092 ± 398 (bc)	5717 ± 547 (ab)	4787 ± 605	2975 ± 546
	[41,50]	2731 ± 176 (ab)	4945 ± 455 (ab)	5737 ± 634 (ab)	4910 ± 647	2998 ± 537
	[51,60]	2743 ± 165 (b)	4886 ± 404 (ab)	5453 ± 565 (ab)	4553 ± 559	2787 ± 351
	[61,70]	2580 ± 174 (a)	4729 ± 382 (a)	5396 ± 387 (a)	4584 ± 549	2903 ± 489
	*p *value	2.341e^−7^	3.993e^−5^	0.0117	0.2053	0.6174
NV (µm^3^)	[20,30]	208 ± 17	155 ± 13	133 ± 6	127 ± 4 (a)	127 ± 7 (a)
	[31,40]	209 ± 13	162 ± 12	137 ± 6	129 ± 4 (ab)	130 ± 7 (ab)
	[41,50]	219 ± 14	169 ± 17	139 ± 9	130 ± 6 (ab)	131 ± 7 (ab)
	[51,60]	208 ± 18	163 ± 19	138 ± 10	130 ± 6 (ab)	132 ± 8 (ab)
	[61,70]	220 ± 28	166 ± 25	138 ± 11	132 ± 6 (b)	135 ± 9 (b)
	*p *value	0.0751	0.1438	0.118	0.0437	0.0373
STD-NV (µm^3^)	[20,30]	96.9 ± 6.2	63.2 ± 7.7 (a)	54.1 ± 6.2	54.7 ± 7.2	59.2 ± 9.2
	[31,40]	95.2 ± 7.2	60.5 ± 4.3 (ab)	53.9 ± 4.2	56.7 ± 5.3	59.7 ± 8.3
	[41,50]	94.6 ± 6.7	63.1 ± 4.3 (ab)	54.3 ± 4.0	55.5 ± 6.9	58.8 ± 8.2
	[51,60]	99.3 ± 7.0	66.0 ± 6.6 (ab)	55.0 ± 4.0	56.0 ± 5.2	60.4 ± 7.1
	[61,70]	96.3 ± 8.7	65.6 ± 8.6 (b)	56.5 ± 6.0	57.7 ± 6.9	61.6 ± 8.7
	*p *value	0.0466	0.0124	0.1272	0.7227	0.6394
NC	[20,30]	0.671 ± 0.029	0.757 ± 0.015 (b)	0.801 ± 0.012	0.810 ± 0.012	0.805 ± 0.014
	[31,40]	0.671 ± 0.031	0.753 ± 0.016 (ab)	0.799 ± 0.010	0.810 ± 0.010	0.805 ± 0.011
	[41,50]	0.659 ± 0.028	0.742 ± 0.021(a)	0.793 ± 0.014	0.809 ± 0.011	0.806 ± 0.014
	[51,60]	0.681 ± 0.028	0.757 ± 0.018 (b)	0.799 ± 0.009	0.810 ± 0.008	0.809 ± 0.009
	[61,70]	0.683 ± 0.026	0.760 ± 0.013 (b)	0.801 ± 0.011	0.814 ± 0.010	0.813 ± 0.010
	*p *value	0.0616	0.012	0.1884	0.5298	0.1867
STD-C	[20,30]	0.106 ± 0.004	0.082 ± 0.004	0.068 ± 0.004	0.066 ± 0.006	0.070 ± 0.007
	[31,40]	0.107 ± 0.005	0.084 ± 0.005	0.068 ± 0.004	0.066 ± 0.004	0.070 + /0.005
	[41,50]	0.107 ± 0.004	0.085 ± 0.007	0.070 ± 0.005	0.067 ± 0.004	0.070 ± 0.006
	[51,60]	0.106 ± 0.005	0.083 ± 0.007	0.069 ± 0.005	0.066 ± 0.004	0.069 ± 0.005
	[61,70]	0.107 ± 0.005	0.083 ± 0.005	0.068 ± 0.005	0.065 ± 0.005	0.067 ± 0.005
	*p *value	0.891	0.309	0.684	0.889	0.411
CNA	[20,30]	0.115 ± 0.017 (a)	0.116 ± 0.025 (a)	0.153 ± 0.028 (a)	0.200 ± 0.039 (a)	0.231 ± 0.027 (a)
	[31,40]	0.124 ± 0.025 (a)	0.129 ± 0.023 (ab)	0.168 ± 0.022 (ab)	0.197 ± 0.020 (a)	0.228 ± 0.022 (a)
	[41,50]	0.130 ± 0.031 (ab)	0.154 ± 0.042 (bc)	0.200 ± 0.049 (b)	0.217 ± 0.038 (ab)	0.240 ± 0.027 (ab)
	[51,60]	0.141 ± 0.030 (ab)	0.153 ± 0.051 (bc)	0.187 ± 0.059 (ab)	0.225 ± 0.043 (ab)	0.249 ± 0.027 (ab)
	[61,70]	0.152 ± 0.044 (b)	0.169 ± 0.057 (c)	0.199 ± 0.048 (b)	0.235 ± 0.035 (b)	0.256 ± 0.035 (b)
	*p *value	0.0028	0.0008	0.0026	0.0047	0.0091

Metrics are presented as mean ± sd, *p* values for each maturation index are provided and letters indicate the outcome from multi-comparison tests (when relevant). CSD, Cell surface density; NV, Nuclei volume; NC, Nuclei compactness; CNA, Cell network atypia; STD, Standard deviation computed as a metric.

**Table 4:** Summary of cellular metrics as function of maturation index (Cheekbone).

Parameters		M5	M4	M3	M2	M1
CSD (cells/mm^2^)	[20,30]	2881 ± 135 (b)	5065 ± 267 (a)	5897 ± 335	5269 ± 344	3347 ± 396
	[31,40]	2891 ± 157 (b)	4992 ± 346 (a)	5896 ± 409	5431 ± 437	3553 ± 528
	[41,50]	2803 ± 171 (ab)	4816 ± 486 (ab)	5813 ± 517	5494 ± 459	3802 ± 581
	[51,60]	2794 ± 166 (ab)	4834 ± 340 (ab)	5813 ± 346	5436 ± 551	3669 ± 695
	[61,70]	2701 ± 167 (a)	4714 ± 316 (b)	5738 ± 418	5497 ± 627	3714 ± 616
	*p *value	0.0018	0.0185	0.7164	0.5584	0.1186
NV(µm^3^)	[20,30]	223 ± 17	176 ± 16	149 ± 10	134 ± 5 (ab)	130 ± 4
	[31,40]	224 ± 19	180 ± 16	149 ± 11	132 ± 5 (a)	129 ± 6
	[41,50]	218 ± 21	176 ± 19	148 ± 13	135 ± 8 (ab)	132 ± 6
	[51,60]	227 ± 24	179 ± 19	151 ± 13	135 ± 6 (ab)	131 ± 5
	[61,70]	236 ± 30	185 ± 24	154 ± 13	139 ± 7 (b)	134 ± 7
	*p *value	0.1237	0.6369	0.6194	0.0279	0.0637
STD-NV (µm^3^)	[20,30]	96.9 ± 6.2 (a)	63.2 ± 7.7 (a)	54.1 ± 6.2 (ab)	54.7 ± 7.2 (ab)	59.2 ± 9.2
	[31,40]	98.8 ± 5.8 (a)	67.3 ± 6.6 (a)	57.4 ± 5.7 (a)	54.8 ± 3.8 (a)	57.3 ± 6.4
	[41,50]	98.7 ± 9.0 (a)	67.1 ± 5.3 (ab)	56.3 ± 4.8 (ab)	53.7 ± 4.3 (ab)	57.7 ± 6.8
	[51,60]	99.2 ± 9.2 (ab)	69.2 ± 7.7 (ab)	58.2 ± 5.4 (ab)	56.9 ± 4.6 (ab)	60.9 ± 78
	[61,70]	101.8 ± 12.4 (b)	69.2 ± 7.1 (b)	58.7 ± 4.6 (b)	56.5 ± 5.1 (b)	59.9 ± 7.1
	*p *value	0.0128	0.0302	0.0396	0.019	0.1858
NC	[20,30]	0.638 ± 0.033	0.735 ± 0.021 (b)	0.793 ± 0.012 (c)	0.813 ± 0.009 (b)	0.814 ± 0.011 (c)
	[31,40]	0.635 ± 0.039	0.727 ± 0.023 (b)	0.789 ± 0.012 (bc)	0.812 ± 0.008 (b)	0.812 ± 0.012 (bc)
	[41,50]	0.632 ± 0.025	0.717 ± 0.018 (ab)	0.775 ± 0.016 (ab)	0.799 ± 0.016 (ab)	0.800 ± 0.018 (a)
	[51,60]	0.631 ± 0.025	0.718 ± 0.020 (ab)	0.778 ± 0.013 (a)	0.803 ± 0.012 (a)	0804 ± 0.015 (abc)
	[61,70]	0.620 ± 0.037	0.706 ± 0.029 (a)	0.768 ± 0.018 (a)	0.797 ± 0.012 (a)	0.802 ± 0.010 (ab)
	*p *value	0.4719	0.0011	6.250e^−7^	1.195e^−5^	0.0032
STD-NC	[20,30]	0.111 ± 0.004	0.090 ± 0.004	0.071 ± 0.004 (a)	0.064 ± 0.004 (a)	0.067 ± 0.006 (a)
	[31,40]	0.111 ± 0.004	0.091 ± 0.006	0.072 ± 0.005 (ab)	0.064 ± 0.003 (a)	0.069 ± 0.007 (ab)
	[41,50]	0.111 ± 0.006	0.092 ± 0.006	0.076 ± 0.006 (abc)	0.069 ± 0.006 (ab)	0.073 ± 0.008 (ab)
	[51,60]	0.111 ± 0.006	0.094 ± 0.006	0.075 ± 0.006 (bc)	0.068 ± 0.005 (b)	0.071 ± 0.007 (b)
	[61,70]	0.111 ± 0.005	0.095 ± 0.007	0.079 ± 0.006 (b)	0.071 ± 0.004 (b)	0.073 ± 0.005 (b)
	*p *value	0.975	0.0901	1.680e^−5^	2.355e^−6^	0.0122
CNA	[20,30]	0.115 ± 0.017 (a)	0.158 ± 0.024 (a)	0.226 ± 0.038 (ab)	0.249 ± 0.037 (ab)	0.245 ± 0.024
	[31,40]	0.116 ± 0.21 (a)	0.152 ± 0.031(a)	0.205 ± 0.039 (a)	0.227 ± 0.033 (a)	0.230 ± 0.021
	[41,50]	0.124 ± 0.030 (ab)	0.173 ± 0.062(ab)	0.235 ± 0.067 (ab)	0.257 ± 0.058 (ab)	0.257 ± 0.059
	[51,60]	0.124 ± 0.024 (ab)	0.169 ± 0.033 (ab)	0.235 ± 0.047 (ab)	0.257 ± 0.041 (ab)	0.248 ± 0.024
	[61,70]	0.143 ± 0.035 (b)	0.203 ± 0.066 (b)	0.267 ± 0.065 (b)	0.279 ± 0.040 (b)	0.260 ± 0.028
	*p *value	0.0086	0.0106	0.0086	0.0067	0.0572

Metrics are presented as mean ± sd, *p* values for each maturation index are provided and letters indicate the outcome from multi-comparison tests (when relevant). CSD, Cell surface density; NV, Nuclei volume; NC, Nuclei compactness; CAN, Cell network atypia; STD, Standard deviation computed as a metric.

**Table 5:** Summary of cellular metrics as function of maturation index (Mandible).

Parameters		M5	M4	M3	M2	M1
CSD (cells/mm^2^)	[20,30]	2787 ± 149 (c)	5276 ± 289 (c)	6135 ± 402 (c)	5213 ± 497 (ab)	3092 ± 537
	[31,40]	2697 ± 156 (bc)	4998 ± 336 (bc)	6027 ± 399 (bc)	5351 ± 530 (ab)	3346 ± 684
	[41,50]	2631 ± 145 (ab)	4831 ± 408 (ab)	6028 ± 401 (bc)	5549 ± 530 (b)	3529 ± 705
	[51,60]	2604 ± 165 (ab)	4642 ± 338 (a)	5569 ± 370 (a)	5058 ± 518 (a)	3254 ± 580
	[61,70]	2519 ± 177 (a)	4560 ± 394 (a)	5686 ± 396 (ab)	5299 ± 448 (ab)	3397 ± 580
	*p *value	*1.23e*^−5^	*1.70e*^−8^	*1.75e*^−5^	*0.044*	*0.243*
NV (µm^3^)	[20,30]	220 ± 17 (a)	165 ± 18 (a)	139 ± 12	129 ± 8	128 ± 7 (ab)
	[31,40]	235 ± 23 (ab)	181 ± 19 (ab)	145 ± 13	130 ± 6	126 ± 7 (a)
	[41,50]	237 ± 16 (ab)	183 ± 16 (ab)	145 ± 10	131 + 6	130 ± 4 (ab)
	[51,60]	225 ± 22 (ab)	180 ± 24 (ab)	145 ± 11	132 ± 5	130 ± 7 (ab)
	[61,70]	245 ± 25 (b)	189 ± 28 (b)	148 ± 15	133 ± 6	132 ± 6 (b)
	*p *value	*0.0019*	*0.0167*	*0.1748*	*0.1634*	*0.0164*
STD-NV (µm^3^)	[20,30]	96.9 ± 6.2 (a)	63.2 ± 7.7 (a)	54.1 ± 6.2 (a)	54.7 ± 7.2 (ab)	59.2 ± 9.2 (a)
	[31,40]	100.0 ± 8.8 (a)	66.9 ± 6.2 (ab)	54.1 ± 4.8 (a)	51.3 ± 3.4 (a)	54.0 ± 6.5 (ab)
	[41,50]	105.8 ± 9.7 (a)	70.9 ± 6.3 (bc)	56.3 ± 4.8 (ab)	55.6 ± 4.1 (ab)	59.8 ± 5.2 (ab)
	[51,60]	101.3 ± 9.6 (ab)	70.8 ± 8.8 (bc)	57.8 ± 4.4 (ab)	56.6 ± 5.9 (b)	60.3 ± 10.3 (ab)
	[61,70]	112.6 ± 17.2 (b)	75.7 ± 11.6 (c)	59.2 ± 6.0 (b)	57.3 ± 5.0 (b)	61.6 ± 6.5 (b)
	*p *value	*0.0002*	*0.0001*	*0.0087*	*0.006*	*0.0295*
NC	[20,30]	0.650 ± 0.027	0.744 ± 0.017 (b)	0.799 ± 0.016 (c)	0.812 ± 0.016	0.808 ± 0.018
	[31,40]	0.644 ± 0.026	0.736 ± 0.016 (ab)	0.793 ± 0.012 (bc)	0.813 ± 0.010	0.813 ± 0.013
	[41,50]	0.642 ± 0.023	0.720 ± 0.028 (a)	0.779 ± 0.019 (a)	0.805 ± 0.010	0.805 ± 0.009
	[51,60]	0.659 ± 0.026	0.734 ± 0.022 (ab)	0.784 ± 0.013 (ab)	0.805 ± 0.010	0.807 ± 0.012
	[61,70]	0.654 ± 0.026	0.730 ± 0.020 (ab)	0.784 ± 0.011 (ab)	0.806 ± 0.008	0.807 ± 0.008
	*p *value	*0.193*	*0.0096*	*0.0002*	*0.037*	*0.317*
STD-C	[20,30]	0.106 ± 0.004	0.084 ± 0.005	0.068 ± 0.005 (a)	0.064 ± 0.006	0.068 ± 0.008
	[31,40]	0.107 ± 0.004	0.086 ± 0.005	0.070 ± 0.004 (ab)	0.064 ± 0.003	0.066 ± 0.006
	[41,50]	0.108 ± 0.005	0.088 ± 0.008	0.074 ± 0.007 (b)	0.067 ± 0.004	0.071 ± 0.005
	[51,60]	0.109 ± 0.008	0.088 ± 0.007	0.074 ± 0.006 (b)	0.068 ± 0.005	0.070 ± 0.007
	[61,70]	0.109 ± 0.007	0.090 ± 0.008	0.074 ± 0.005 (b)	0.067 ± 0.003	0.070 ± 0.004
	*p *value	*0.392*	*0.083*	*0.021*	*0.0101*	*0.0537*
*CNA*	[20,30]	0.106 ± 0.017 (a)	0.123 ± 0.028 (a)	0.169 ± 0.044 (a)	0.205 ± 0.044 (a)	0.233 ± 0.036
	[31,40]	0.117 ± 0.023 (a)	0.154 ± 0.043 (ab)	0.196 ± 0.048 (ab)	0.205 ± 0.037 (a)	0.222 ± 0.025
	[41,50]	0.128 ± 0.024 (ab)	0.167 ± 0.055 (ab)	0.203 ± 0.062 (ab)	0.225 ± 0.042 (ab)	0.237 ± 0.023
	[51,60]	0.139 ± 0.030 (ab)	0.176 ± 0.056 (b)	0.218 ± 0.057 (ab)	0.229 ± 0.045 (ab)	0.236 ± 0.027
	[61,70]	0.162 ± 0.074 (b)	0.204 ± 0.086 (b)	0.241 ± 0.073 (b)	0.243 ± 0.043 (b)	0.241 ± 0.018
	*p* value	*0.0003*	*0.0006*	*0.0240*	*0.0221*	*0.1935*

Metrics are presented as mean ± sd, *p* values for each maturation index are provided and letters indicate the outcome from multi-comparison tests (when relevant). CSD, Cell surface density; NV, Nuclei volume; NC, Nuclei compactness; CAN, Cell network atypia; STD, Standard deviation computed as a metric.

First reported in the literature in 2018^18^, LC-OCT is an emerging technique that, coupled with AI based segmentation algorithms, has proven its capabilities to characterise skin structures to extract histological^20,27^ and cellular information^24,25^. Over the years, skin microanatomy has been investigated quite extensively based on histological data which provided initial knowledge of physiological processes. However it is the improvements in in vivo imaging techniques that have enabled to access information at cellular level. Notably, microscopy investigations linked smaller keratinocytes in infants to higher cell turnover^50^, followed by an increase in size and a decrease in cell density with age, both in the *stratum granulosum* and *stratum spinosum*^51^. Presently, cellular metrics computed from LC-OCT images corroborated these observations with significant decrease in CSD for the temple, cheekbone and mandible with age but also an increase in the number of cell layers for the mandible and an increase in the standard deviation of number of cell layers for the temple and mandible for younger age groups, that could be linked to higher proliferating activities. Historically, RCM has been widely used for the measurement of keratinocyte size at several different depths^13^ and the honeycomb pattern formed by these cells has been described^52^. While RCM was limited to 2D horizontal images for in vivo morphometric analyses^30^, it has been largely used to characterise, for example, photoageing comparing sun-exposed and sun-protected body sites^30^. Indeed, improved resolution or, for instance, direct access to 3D acquisition in recent technologies, coupled with more robust data analysis protocols have made it possible to compute cellular parameters (size, shape)^53^. LC-OCT presents relevant capabilities, positioning it as a valuable complementary tool for performing multiparametric quantification of skin aging through automated determination of metrics at the micrometric level. The technique benefits from a substantial field of view (1200 × 500 µm^2^) and a depth of analysis spanning 500 µm. Notably, the highly isotropic resolution of approximately 1.3 µm enhances its performance by enabling precise three-dimensional imaging, which can provide valuable insights into skin physiological processes, skin biology, and the aging process. The performances to visualise fine structures of human skin in vivo are comparable to that of multiphoton microscopy which has witnessed drastic improvement for investigation of keratinocytes morphology in vivo^54^ and for the 3D quantification of human skin ageing^39^. Both MPLSM and LC-OCT are optical techniques relying on different modalities that make the information collected highly complementary. Nevertheless, similar to other imaging techniques, using 3D acquisitions in this context entails two main challenges. First, the large datasets and the complexity of biological information require the development of sophisticated data mining methods^55^. Second, the analysis time is influenced by several factors, including the time required to collect a single 3D image (usually less than a minute), the number of images needed for representative and reliable results from a specific area (a few minutes), and the number of facial areas to be analysed to obtain comprehensive information for the entire face (tens of minutes). While the combination of micrometric resolution and full-face in vivo microscopy represents a substantial technical challenge, recent advancements in imaging systems and computational techniques, particularly the integration of LC-OCT imaging with AI-based algorithms, hold promise for future real-time 3D visualisation of skin structures in the most relevant facial areas for aging investigations. Achieving this milestone would mark a significant advancement in the non-invasive characterisation of healthy skin.

## Conclusion

The potential of in vivo 3D LC-OCT imaging to generate large datasets from extensive panels of healthy volunteers, currently comprising 100 female Caucasians, has been demonstrated. The technique high isotropic resolution and depth of analysis facilitated the computation of histological and cellular epidermal quantitative metrics using AI-based algorithms, enabling the correlation of skin microstructure variations with ageing. This initial exploratory study highlighted the technique capabilities in supporting a better understanding of the ageing process, particularly in determining skin layer thickness with micrometric precision at different facial anatomical sites (temple, cheekbone, and mandible). Additionally, the study provides further insights into the slight thickening of the stratum corneum (temple and mandible) and consistent viable epidermis thickness (temple, cheekbone) with ageing, contributing valuable data to the existing literature. Furthermore, at the cellular level, 3D LC-OCT proves to be a powerful tool for assessing the keratinocyte network. The observed decreased standard deviation in the number of cell layers, reduced cell surface density, increased nuclei mean volume, higher standard deviation for nucleus volume, and increased cell network atypia collectively indicate a less dense cellular network with a reduced number of cells per layer. Additionally, the presence of larger and more heterogeneous nuclei is associated with ageing. Coupling in vivo 3D LC-OCT imaging with AI represents a valuable tool to gain insights into the microstructural changes occurring in the skin with advancing age. Additionally, the derived cellular metrics hold promises as potential key biomarkers to quantify facial skin ageing in healthy Caucasian female volunteers.

## Materials and methods

### Study population

The research was conducted performed in accordance with local (FRANCE) legal regulations and in accordance with the Declaration of Helsinki. The study was approved by the French ethical committee “Comité de protection des personnes sud méditerranée I” (IDRCB : 2021-A00101-40). A written informed consent was obtained from all participants. One hundred healthy Caucasian female volunteers with I, II or III skin phototype (Fitzpatrick scale) and evenly distributed in 5 age groups [20,30], [31,40], [41,50], [51,60] and [61,70] were included.

### LC-OCT^®^ in vivo 3D imaging

**Figure 1:**
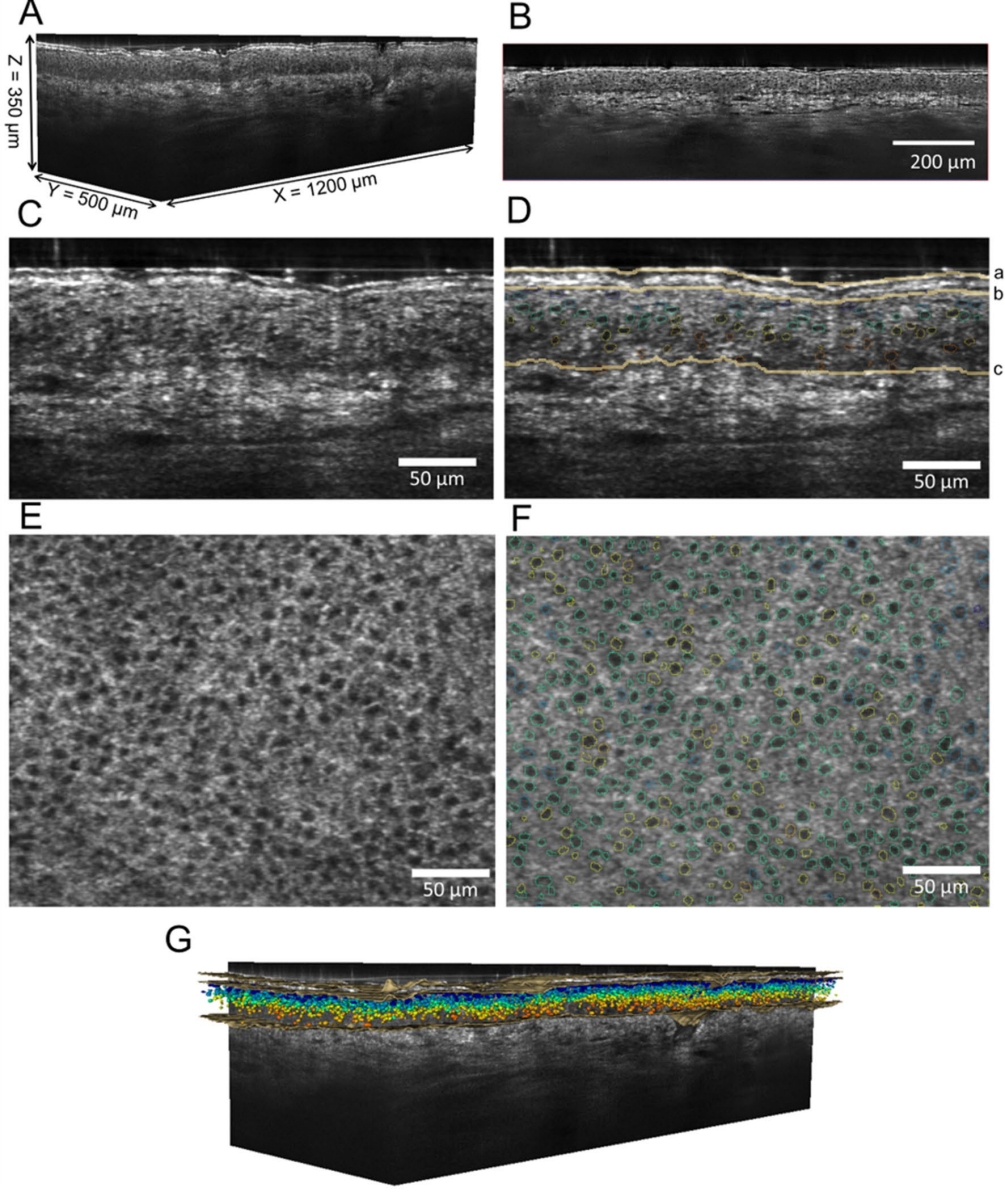
LC-OCT coupled to AI applied to imaging of healthy human face skin. (**A**) Example of 3D stack acquired. (**B**) Representative 2D reconstructed vertical image (xz). (**C**,**D**) illustration of a 2D reconstructed vertical image (enlarged view) before and after the segmentation of layers (a: Skin surface, b: SC-Viable epidermis interface and c: Epidermis-Dermis interface (i.e. dermal–epidermal junction). (**E**,**F**): Illustration of 2D horizontal image (xy, enlarged view) before and after cell nuclei segmentation (coloured circular shaped lines represent nuclei detected within these stacks from 3D LC-OCT images). (**G**) LC-OCT images (3D stack) with colour projection of layers and cellular metrics.

3D images were collected using a deepLive™ system (DAMAE MEDICAL, France) (Fig. 1A). The instrumental setup is detailed in^56^. Paraffin oil (n ∼ 1.4) was used as immersion medium. Three LC-OCT 3D images (1200 µm × 500 µm × 350 µm) were acquired for each area (temple, cheekbone and mandible (inferior jawline)) as stacks of slices parallel to the skin surface (xy) with a 1 μm step size (z direction). The side of the face analysed for each subject was defined by a randomisation method. To ensure the representativeness of the sampling, visual quality criteria were applied, which included no movement during acquisition, maintaining proper contact of the probe with the skin, minimising the presence of hairs and skin appendices, and ensuring the absence of pigmented spots in the area of interest.

### LC-OCT^®^ images analysis

#### Thickness of layers and DEJ segmentation

Skin layer segmentations was performed using a 2D UNet model on vertical reconstructed images (Fig. 1B)^57,58^. The algorithm was trained using manually annotated 2D vertical images labelled by trained experts from an independent dataset. The segmentation outcome was also validated by the trained experts through visual inspection of processed images to ensure accuracy and reliability. Thickness of the SC and VE were derived from the average numbers of pixels between the skin surface, the SC interface and the DEJ interface, respectively (Fig. 1B).

The percentage of DEJ undulation was calculated as follow:

$$\% {\text{U}}_{{{\text{DEJ}}}} = \left( {\left( {{\text{S}}_{{{\text{DEJ}}}} /{\text{S}}_{{{\text{ROI}}}} } \right) - {1}} \right) \times {1}00,$$

where S_DEJ_ is the area of the dermo-epidermal interface and S_ROI_ the total horizontal area of the LC-OCT image excluding areas corresponding to hair follicles^24,59^.

#### Cellular metrics

Segmentation of cells nuclei in the VE was performed by deep learning models (AI algorithm) based on 3D convolutions^24^ and the 3D StarDist model^60^ (Fig. 1C and D). Additional details are provided in^25^. The development of the method was supported by trained experts who manually annotated 2D vertical images and validated the outcome of the nuclei segmentation trough thorough visual inspection. Cell surface density (cell number/mm^2^), volume (μm^3^), compactness (or sphericity, number of cell layers (in the VE, Fig. 1C) and nuclei network atypia (detection of outliers in the cell population taking into account their position) were computed. More details about the model used for nuclei network atypia (XGBoost^61^) can be found in^25^. To reflect the intra-image variability standard deviation of nuclei volume, standard deviation of nuclei compactness and standard deviation of the number of cell layers were also used as metrics.

#### Cellular maturation

To account for the biological variations associated with the maturation of keratinocytes across the *Stratum Basale*, *Stratum Spinosum*, and *Stratum Granulosum* layers, cellular metrics were analysed based on their spatial distribution within the depth of the VE (Fig. 1G). To standardize the positioning across all LC-OCT images, the cellular metrics were subdivided into five segments, which can be assimilated to quintiles used in statistics. Each segment was labelled with a maturation index ranging from M1 (the deepest segment corresponding to the lower 20% fraction of the VE and associated with the *Stratum Basale*) to M5 (the upper segment corresponding to the higher 20% fraction of the VE associated with the *Stratum Granulosum*). Thus, each maturation index encompasses changes in the cellular network (nuclei size and shape) correlated with the differentiation of keratinocytes.

#### Statistical analysis

The metrics were reported as mean ± standard deviation. An ANOVA was carried out in order to compare the subjects according to the age groups. The normality of the ANOVA residues was assessed by a Shapiro–Wilk test, with a significance level set at 10%. In the event that the normality of the residuals was not verified, a Kruskal–Wallis test was used as an alternative. When ANOVA (or KW test) was significant, multi-comparison tests were performed to compare age groups pairwise, with Tukey adjustment for multiple comparisons tests. The letters assigned to each age group illustrate their discrimination and to visualise significant variations in metrics with age.

### Supplementary Information

Supplementary Figures.

## Data Availability

All data are available in the text.
