# Supplementary material for: Line-field confocal optical coherence tomography coupled with artificial intelligence algorithms to identify quantitative biomarkers of facial skin ageing
Source: Sci Rep. 2023 Aug 24;13:13881. doi: 10.1038/s41598-023-40340-0 (PMC10449778; doi:10.1038/s41598-023-40340-0)
Supplement: Supplementary file 1 — Supplementary Figures. [file 41598_2023_40340_MOESM1_ESM.docx]

**Line-field confocal optical coherence tomography coupled with artificial intelligence algorithms to identify quantitative biomarkers of facial skin ageing**

**Bonnier, Franck^1,*^**; Pedrazzani, Mélanie^2^; Fischman, Sébastien^2^; Viel, Théo^2^; Lavoix, Agnes^3^; Pegoud, Didier^3^; Nili, Meryem^3^; Jimenez, Yolande^3^; Ralambondrainy, Samuel^1^; Cauchard Jean-Hubert^1^; Korichi, Rodolphe^1^

^1^ *LVMH Recherche, 185 avenue de Verdun, 45804 Saint Jean de Braye, Franc*e

*^2^ DAMAE Medical, 14 Rue Sthrau, 75013 Paris, France*

*^3^ DERMATECH, 8 Rue Jacqueline Auriol, 69008 Lyon*

*** Bonnier Franck, 185 avenue de Verdun, 45804 Saint Jean de Braye, France, +33 2 38 60 31 32, [fbonnier@research.lvmh-pc.com](mailto:fbonnier@research.lvmh-pc.com)

**SUPPLEMENTARY DATA**


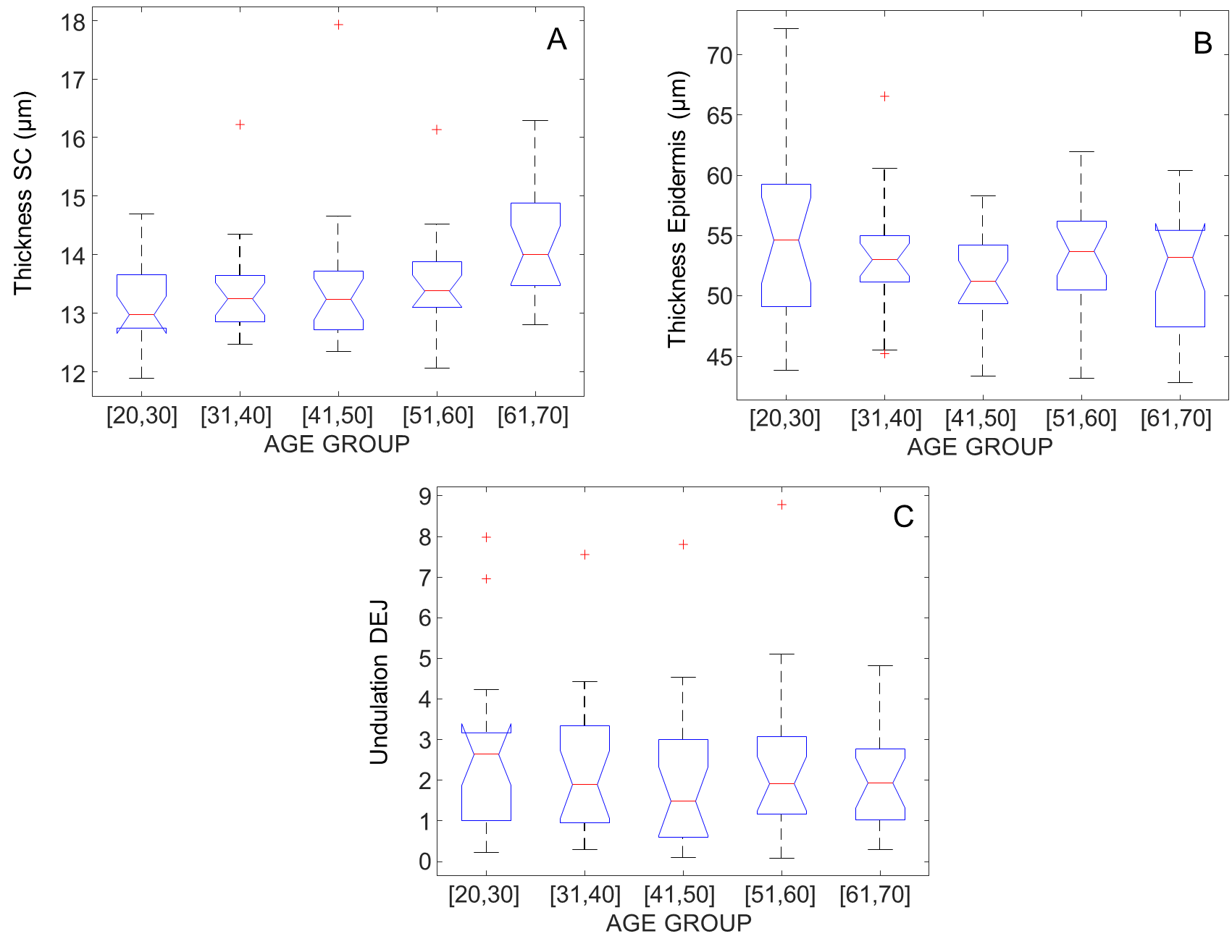


Figure S1: Characterisation of age-related variations in skin layers for the temple. Boxplots for the thickness of the *stratum corneum* (A), the thickness of viable epidermis (B) and Undulation of the DEJ (C).


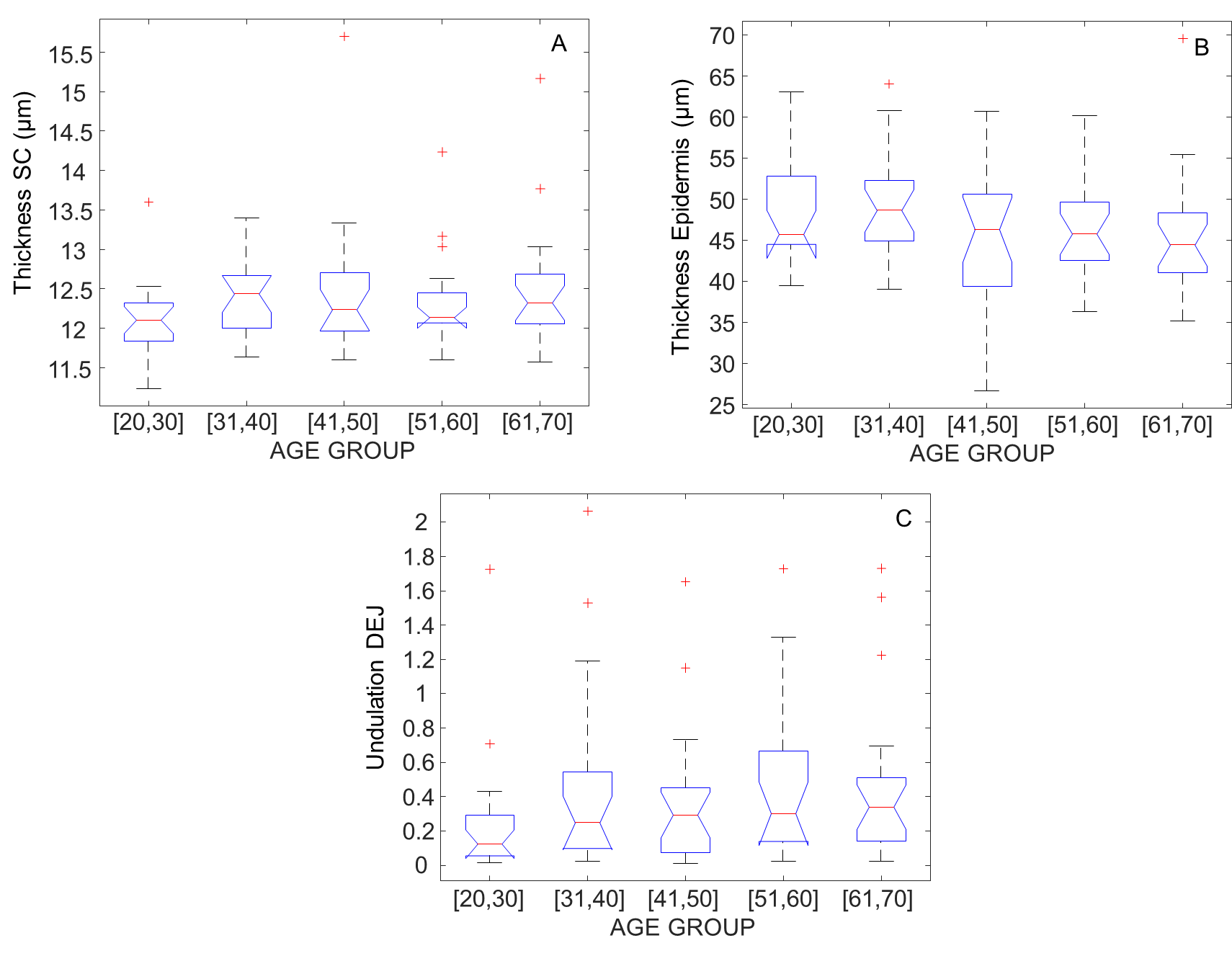


Figure S2: Characterisation of age-related variations in skin layers for the cheekbone. Boxplots for the thickness of the *stratum corneum* (A), the thickness of viable epidermis (B) and Undulation of the DEJ (C).


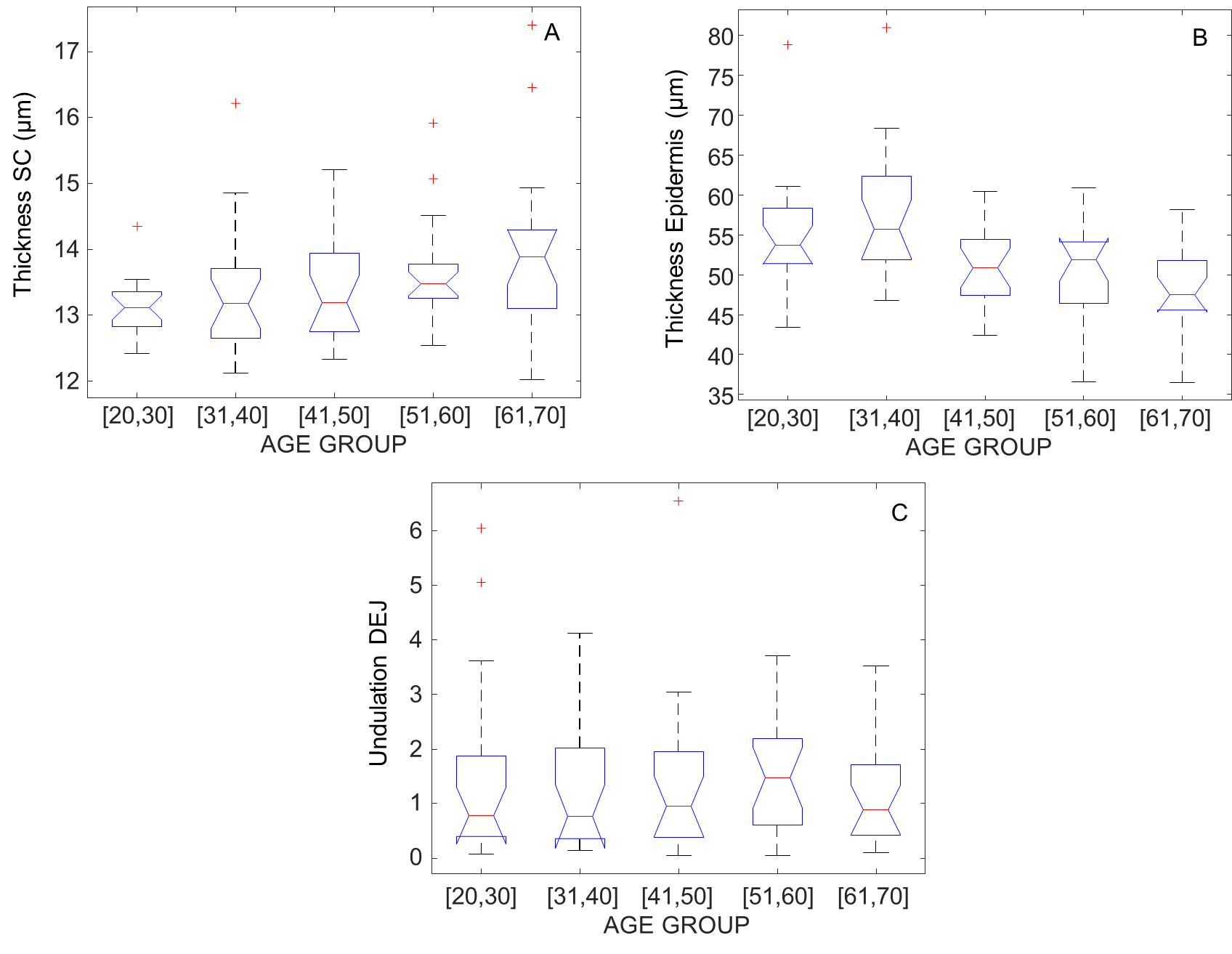


Figure S3: Characterisation of age-related variations in skin layers for the mandible. Boxplots for the thickness of the stratum corneum (A), the thickness of viable epidermis (B) and Undulation of the DEJ (C).
